# The Role of BCL2 Family of Apoptosis Regulator Proteins in Acute and Chronic Leukemias

**DOI:** 10.1155/2012/524308

**Published:** 2011-09-14

**Authors:** Flora Tzifi, Christina Economopoulou, Dimitrios Gourgiotis, Alexandros Ardavanis, Sotirios Papageorgiou, Andreas Scorilas

**Affiliations:** ^1^Department of Biochemistry and Molecular Biology, University of Athens, Panepistimiopolis, 15701 Athens, Greece; ^2^“Aghia Sophia” Children's Hospital, Thibon & Papadiamantopoulou, 11527 Athens, Greece; ^3^Second Department of Internal Medicine—Propaedeutic, Hematology Unit, University of Athens, Medical School, University General Hospital “Attikon”, 1 Rimini Street, 12462 Haidari, Greece; ^4^Research Laboratories, 2nd Department of Pediatrics, Medical School, University of Athens, P & A Kyriakou Children's Hospital Thivon & Levadeias, 11527 Athens, Greece; ^5^First Department of Oncology, St. Savvas Anticancer Hospital, 171, Alexandras Avenue, 11522 Athens, Greece

## Abstract

The disturbance of apoptosis molecular signaling pathways is involved in carcinogenesis. BCL2 family of proteins is the hallmark of apoptosis regulation. In the last decade, new members of *BCL2* gene family were discovered and cloned and were found to be differentially expressed in many types of cancer. BCL2 protein family, through its role in regulation of apoptotic pathways, is possibly related to cancer pathophysiology and resistance to conventional chemotherapy. It is well known that leukemias are haematopoietic malignancies characterized by biological diversity, varied cytogenetics, different immunophenotype profiles, and diverse outcome. Current research focuses on the prognostic impact and specific role of these proteins in the pathogenesis of leukemias. The understanding of the molecular pathways that participate in the biology of leukemias may lead to the design of new therapies which may improve patients' survival. In the present paper, we describe current knowledge on the role of BCL2 apoptosis regulator proteins in acute and chronic leukemias.

## 1. Introduction

Apoptosis, or programmed cell death, is a cell-suicide program, distinct from necrosis, which is activated in physiological processes such as tissue development and differentiation as well as in pathophysiological conditions. The term is used to describe the situation in which a cell actively pursues a course toward death upon receiving certain stimuli. The morphological changes of apoptosis found in most cell types include nuclear pyknosis, DNA fragmentation and chromatin condensation, cytoskeleton destruction, membrane blebbing, and eventually the formation of membrane apoptotic bodies, which are phagocytosed by macrophages and other cells without promoting inflammatory response [1]. The mechanism of apoptosis is evolutionarily conserved and is executed by a family of proteins, called caspases. Caspases are cysteine proteases that are cleaved after an Asp residue in their substrates. They are synthesized as latent zymogenes and activated by proteolytic cleavage; their activation is mainly regulated by the BCL2 family proteins [2–4].

*BCL2* gene (otherwise B-cell lymphoma 2 gene, bcl-2) was first discovered in follicular B-cell lymphoma as a gene which is linked to the immunoglobulin heavy chain locus at the breakpoints of t(14;18) translocation [5]; the result of this translocation is the enhanced BCL2 protein transcription. In normal cells this gene is located on chromosome segment 18q21.3. BCL2 protein was found to inhibit cell death. This discovery was a revolution in the way of approaching cancer pathology, since it gave birth to the notion that tumor genesis could be due to not only unlimited proliferation, but also to impaired apoptosis. It should be noticed that BCL2 oncoprotein overexpression is found not only in follicular non-Hodgkin's lymphoma but also in other haematopoietic malignancies and solid tumors, independent of t(14;18) chromosomal translocation.

**Figure 1:**
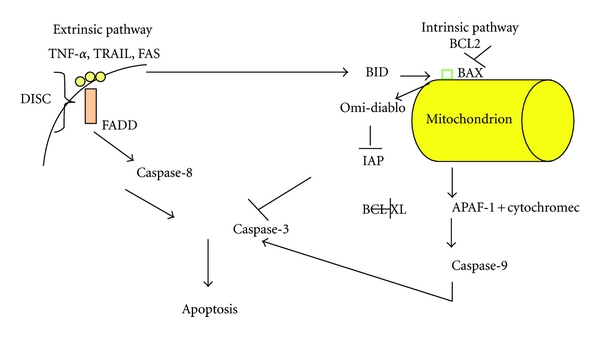
In this figure we show the two main pathways to apoptosis, and their interaction through the molecule BID, since death receptors activate the intrinsic pathway by activating BID. Either BAX or BAK are required for apoptosis, where they oligomerize in the mitochondrial outer membrane and induce the release of cytochrome c. DISC: death-inducing signaling complex, FADD: FAS-associated protein with death domain, TRAIL: TNF-related apoptosis-inducing ligand, APAF-1: apoptotic protease-activating factor, IAP: inhibitor of apoptosis proteins.

There are two known distinct pathways which lead to apoptosis (Figure 1). The first, which is called the intrinsic cell death pathway, is evoked by intracellular stresses like radiation, growth factor withdrawal, cytokine deprivation, cytotoxic drugs and is regulated by BCL2 family proteins [6, 7]. Progression through this pathway leads to the release of cytochrome c from the damaged mitochondrion, which then binds to the adaptor molecule APAF-1 and an inactive “initiator” caspase, procaspase 9, within a multiprotein complex called the apoptosome. This leads to the activation of caspase 9, which then triggers a cascade of caspases activation (caspases 3 and 7) resulting in the morphological and biochemical changes associated with apoptosis. The second cell death pathway is the extrinsic pathway, which functions independently of mitochondria. This pathway is activated by the cell-surface death receptors CD95 (Apo-1 or Fas)/TRAIL/tumor necrosis factor (TNF) receptor 1 family proteins which are located on the plasma membrane, and directly activates the caspase cascade via the recruitment of the “initiator” caspase-8 within a death-inducing signaling complex (DISC) [8].

Impaired apoptosis is a hallmark of the pathogenesis of many forms of cancer [9–16]. This paper focuses on the role of BCL2 family members in the biology, progression, prognosis, and therapy of acute and chronic leukemias.

## 2. BCL2 Family of Apoptosis Regulator Proteins

Mammalian BCL2 protein family consists of at least 30 related proteins, characterized by the presence of up to four relatively short sequence motifs (less than 20 amino acid residues in length) termed BCL2 homology (BH) domains [17–21]. BCL2 family is divided into three different subclasses based on structural and functional features.

### 2.1. Prosurvival or Antiapoptotic Family Members

The prosurvival or antiapoptotic subfamily includes BCL2, BCL-XL, BCL-W, and MCL-1 proteins, which possess all four conserved BH domains, designated BH1-4, and a hydrophobic C terminal part. BH1-BH3 domains form a hydrophobic groove and the N-terminal BH4 domain stabilizes this structure. BH4 domain is usually absent in apoptotic proteins and therefore is a key factor for the antiapoptotic activity. The BH4 domain of BCL2 consists of 26 amino acids and its structure shows an amphipathic character upon interaction with the membranes, akin to antimicrobial peptides. The *β*-sheet conformation of BH4 in water is concerted into an a-helical structure, appropriate to interact favorably with the negatively charged membranes [22]. It appears that the proteins are mainly located outside the membrane, however their exact insertion and complex formation is not well understood. BCL2 is permanently found in membranes, whereas BCL-XL and BCL-W are linked to the membrane after a cytotoxic signal [6].

BCL2 (and its antiapoptotic orthologues) seems to inhibit apoptosis by the preservation of mitochondrial membrane integrity as its hydrophobic carboxyl-terminal domain is linked to the outer membrane. BCL2 prevents BAX/BAK oligomerization, which would otherwise lead to the release of several apoptogenic molecules from the mitochondrion. It is also known that BCL2 binds to and inactivates BAX and other pro-apoptotic proteins, thereby inhibiting apoptosis. BCL2 might also regulate the activation of several initiator caspases like caspase-2 that act upstream or independently of cytochrome c release from mitochondria. Moreover, BCL2 directly blocks cytochrome c release and therefore prevents APAF-1 and caspase-9 activation.

BCL2 has not only been localized to the outer mitochondrial membrane but also to the nuclear envelope and the endoplastic reticulum membrane (ER). In the ER, it regulates calcium storage, whose intracellular levels have been shown to affect apoptosis. ER-associated BCL2 is able to protect from apoptosis induced by various triggers. Beyond BCL2, BCL-XL also interacts with pro-apoptotic members like BAX and BAK thought their BH3 domains [22, 23]. It is possible that the antiapoptotic action of BCL2 and BCL-XL is converted to a pro-apoptotic one when these proteins are cleaved by caspases after initiation of apoptosis [24].

MCL-1 protein has a short half-life (estimated at less than 1 h) which is unique among antiapoptotic BCL-2 family members. Under basal conditions, human MCL-1 undergoes rapid protein turnover, but the control of this constitutive degradation pathway is incompletely understood. MCL-1 can be cleaved by caspases and granzyme B, which proteolytically degrade MCL-1 during cell death. In addition, human MCL-1 can be ubiquitinylated and degraded by the proteasome. Several levels of degradation control have been postulated. The tight regulation of MCL-1 protein expression makes it an ideal regulator of cell survival. In response to cellular signaling, MCL-1 protein levels can be rapidly induced by inducing new MCL-1 transcription and by preventing MCL-1 protein turnover. When cells need to be eliminated, MCL-1 levels can be rapidly diminished by blocking new protein synthesis and degrading the existing MCL-1. Dysregulation of this balance, by inappropriately promoting its synthesis or by blocking its elimination, can lead to inappropriate stabilization of MCL-1 and promote cellular survival. Furthermore, dysregulated MCL-1 levels can lead to inappropriate cell survival or death; therefore, understanding regulation of MCL-1 levels is of great importance [25–27].

### 2.2. Proapoptotic Family Members

The pro-apoptotic members such as BAX, BAK, and BOK usually share sequence similarity in BH1, BH2, BH3 but not in BH4 domain [17, 28–30]. BAX protein is a monomeric protein in the cytosol, which integrates into the mitochondria during apoptosis and subsequently oligomerizes, resulting *to* the release of apoptogenic factors like cytochrome c and the activation of the caspase cascade. On the other hand, BAK is an integral mitochondrial membrane protein, which also undergoes conformational changes to form larger aggregates during apoptosis [29]. BAX and BAK are also present in the ER, where they control apoptosis through the regulation of calcium levels [31]. The localization of BCL2 to the ER membrane supports the concept that it regulates ER-located BAX and BAK function. P53 can activate BAX, which lacks a clearly identifiable BH3 domain [32]. During drug-induced apoptosis BAX is cleaved by caspases and activated calpains [33].

### 2.3. BH3-Only Family Members

BH3-only proteins include a number of the proapoptotic proteins such as BID, BIM, BIK, BAD, BMF, HRK, DIVA, NOXA, and PUMA. These share sequence similarity only in the BH3 domain. They are activated by different mechanisms, among which are transcriptional upregulation, proteolytic truncation, and phosphorylation. BIM and BMF are released upon phosphorylation, whereas BAD is released upon dephosphorylation [34]. BAD molecule is also cleaved by caspases when apoptosis is induced by transforming growth factor *β*1 [35]. BID is unique among the BH3-only BCL2 family members in interconnecting death receptors to the mitochondrial amplification loop of the intrinsic pathway. The potent proapoptotic activity and broad expression patterns of BID require that cells carefully regulate its apoptotic activation. Subcellular localization appears to play a role in directing the proapoptotic activity of BID. Following death receptor stimulation, BID is activated by caspase-8 cleavage and N-myristoylation to target mitochondria, where it activates BAX and BAK or is alternatively sequestered by antiapoptotic BCL2 members, preventing death. Full-length BID is also capable of translocation to the mitochondria in at least one case facilitated by other proteins such as PACS2. In the mitochondria, full-length BID has been shown to potentiate cell death following certain apoptotic signals, suggesting that caspase cleavage is not an absolute requirement for activating the proapoptotic function of BID. Recent studies indicate that activation of prodeath activity of BID may be negatively regulated by phosphorylation. Casein kinases have been implicated in BID phosphorylation, and ATM has been shown to phosphorylate BID following DNA damage [36–39]. Moreover, BID is proteolytically cleaved by caspase-8 and granzyme B. In acute T lymphoblastic leukemia cells, BID was found to be proteolytically activated by an aspartate-specific protease and to play a crucial role in mitochondrial activation in the p53-independent DNA damage response to etoposide and *γ*-radiation [40]. BH3-only proteins have dual function: both positive and negative regulation of BCL2 family members. Several BH3 peptides relieved the inhibition of BAX caused by the antiapoptotic BCL-XL and/or MCL-1 proteins and some of them display specificity for either BCL-XL or MCL-1. BIM and BID were the only BH3 peptides found to induce cytochrome c release from mitochondria in vitro. They are thought to trigger apoptosis by binding and therefore inactivating the antiapoptotic BCL2 relatives, whereas BID seems to promote apoptosis by activating BAX and BAK [41]. Recent studies show that there are two different subgroups in the BH3-only proteins: the first consists of BIM and BID which induce mitochondria permeabilization via BAX and BAK and are called death agonists. The second includes BAD and BIK. These proteins induce mitochondria permeabilization by opposing antiapoptotic proteins like BCL2, and they are called survival agonists. Peptides that resemble BIM and BID can directly activate BAD, whereas the rest BH3-domain-like peptides act indirectly and require the presence of BIM and/or BID that can directly activate BAD [41].

### 2.4. Newly Identified Proteins of the BCL2 Family Include BCL2L10 (BOO/DIVA), BCL2L12, BCL2L13 (BCL-RAMBO), BCL2L14 (BCL-G), and MAP-1 [9]

*BCL2L10* is an antiapoptotic gene mapped on human chromosome 15q21.2. It encodes for the widely expressed protein BCL2L10 (BOO/DIVA) in adult human tissues, with its highest levels typically found in liver, pancreas, kidneys, brain, and lungs. 

*BCL2L12* gene maps on chromosome 19q13.3. It encodes for BCL2L12 protein which has a predominant molecular mass of 36.8 kDa. BCL2L12 protein contains the conserved BH2 domain of BCL2 family and a putative BH3 domain [9, 10]. There is evidence that BCL2L12 interacts with BCL-X_L_ protein. Additionally, it bears repeated PXXP motifs and a proline rich region that is essential for the interaction with the src homology region (SH3) of tyrosine kinases, such as the protooncogenes c-Scr and c-Abl. It is worth mentioning that it is the first gene identified encoding for a protein which contains both a proline rich and a BH2 domain. The recent identification of the BAX-binding protein BIF-1 suggests a probable connecting role of BCL2L12 among the apoptotic proteins and the SH3-bearing oncoproteins [9, 10]. Recently, it was found that BCL2L12 neutralizes p53 signaling in glioblastoma [8]. One splicing variant missing exon 3 and expressing a 176 amino acid truncated protein with no BH2 homology domain has also been identified. The classic form of the BCL2L12 protein is highly expressed in the thymus, prostate, fetal liver, mammary, colon, placenta, small intestine, kidney, and bone marrow, with lower levels being expressed in all other tissues. The splice variant is highly expressed in fetal liver, spinal cord, and skeletal muscle, where it is present at higher levels than the classical form of the gene, compared to the other tissues. BCL2L12 is also overexpressed in many types of malignancies [9–13].

BCL2L13 widely expressed protein displays a significant similarity to the BCL2 family of proteins, containing all four conserved BH domains (BH1/BH2/BH3/BH4), separated by a 250 amino acid insertion with two tandem repeats rich in serine residues from the characteristic hydrophobic c-terminal membrane anchor (MA). It is characterized by proapoptotic activity and is localized to mitochondria in mammalian cells, although it appears that it induces apoptosis independently of the classical mitochondrial signaling pathways without involving BH4 or other BH domains.

BCL2L14 is another novel human proapoptotic member of the BCL2 protein family. The *BCL2L14* gene maps on chromosome 12p12. It consists of six exons and undergoes alternative splicing producing three different proteins (BCL-G_s_, BCL-G_M_, BCL-G_L_), whose overexpression in various cell lines, such as COS-7 and HEK293T, induces apoptosis. The largest product, BCL-G_L_ (327 amino acids), is diffusely distributed in the cytosol and displays a wide tissue distribution including bone marrow, prostate, pancreas, colon, testis, and spleen. It possesses both BH2 and BH3 domains and it can interact with BCL-X_L_, which blocks its proapoptotic function.

MAP-1 (modulator of apoptosis-1) is another proapoptotic BH3 domain-only protein. It interacts with BAX, BCL2, and BCL-X_L_ and itself to form dimers in vivo and in vitro in mammalian cells. Its association with BAX through its BH3 motif seems to be responsible for its caspase-dependent proapoptotic function, which is evident upon overexpression [9]. The better studied members of *BCL2* gene family are further presented in Table 2.

## 3. BCL2 Gene Family and ALL

A number of studies have linked impaired apoptosis and de-regulation of BCL2 gene family with the pathogenesis and treatment failure in ALL. A recent study [42] indicated a high frequency of *BCL2* mRNA overexpression and a relatively low frequency of *BAX* mRNA overexpression in ALL and AML, suggesting that altered transcription of these genes may be involved in leukemogenesis. Moreover, *BCL2* expression in neoplastic cells from patients with precursor B-ALL, typical ALL and atypical ALL was found to be aberrant in 84%, 77%, and 75% of the cases, respectively, consistent with a diverse expression of BCL2 in the different types of ALL according to the stage of B-cell maturation. [43]. In other words, abnormal BCL2 gene expression seems to influence the survival capacity of B-cell progenitors and contribute to leukemogenesis [44]. Additionally, Aref et al. [45] showed that the expression of *BCL2* was higher in patients with ALL as compared to controls. Although there is a higher expression of *BCL2* in ALL patients, clinical studies failed to correlate this with survival. Sahu and Das [46] found that there was no correlation between *BCL2* expression and overall survival. Another study by Campos et al. led to similar results: high levels of *BCL2* were not associated with clinical or biological characteristics in adult patients with ALL (survival of leukemic cells, outcome after intensive chemotherapy) [47]. Although ALL patients that responded to induction chemotherapy had lower *BCL2* expression compared to the nonresponders, no correlation between *BCL2* expression and the outcome was found.

Findings regarding lineage-dependent *BCL2* expression in ALL are controversial. According to one study, T-ALL but not B-ALL blasts showed higher BCL2 expression in comparison to normal subjects. This finding could explain the poor outcome of the adult patients with T-ALL. On the other hand, another study showed that blasts from pediatric patients with T-ALL expressed lower BCL2 protein when compared to patients with B-ALL [48]. Recent data from more sophisticated techniques, such as DNA microarrays, are also informative of the role of apoptosis genes in ALL. The expression of apoptosis genes is different in the subtypes of ALL, according to lineage origin of the disease and the cytogenetic features. As far as other clinical features are concerned, CD10 positive B-ALL blasts produce higher levels of BCL2 and there is no correlation between BAX expression or BCL2/BAX ratio and other prognostic features of ALL like age, gender, karyotype, or WBC count at the time of diagnosis. Finally, lower expression of BCL2 protein among patients with ALL is observed in patients older than 45 years old and patients with an abnormal karyotype, that is, chromosome of Philadelphia or other translocations [49, 50]. 

The molecular events underlying the progression of T-lymphoblastic lymphoma (T-LBL) to acute T-lymphoblastic leukemia (T-ALL) remain elusive. A recent study revealed autophagy and increased levels of BCL2, S1P1, and ICAM1 in human T-LBL compared with T-ALL. Inhibition of S1P1 signaling in T-LBL cells led to decreased homotypic adhesion in vitro and increased tumor cell intravasation in vivo [51].

As far as other members of the BCL2 family are concerned, there are data concerning BAX and BCLXL that are worth mentioning. Studies investigating the expression of BAX protein and the probability of relapse in children with ALL are contradictive. High levels of BAX protein have been associated with an increased probability of relapse [48]. However, both BAX expression levels and the BAX/BCL2 ratio were, according to another study, significantly lower in samples at relapse compared to samples at initial diagnosis. Moreover, at initial diagnosis ALL patients displayed spontaneous in vivo processing of caspase 3, whereas this was completely absent at relapse [52]. BCLXL has been shown in animal studies to demonstrate an oncogenic synergy with the c-myc oncogene towards the development of ALL [53], and in ALL pediatric patients it could represent an independent prognostic factor of overall survival [54].

Furthermore, BCL2 levels influence the sensitivity of leukemic cells to therapy [44]. According to a recent study, there is an association between lower expression levels of *CASP3, CASP8*, and *FAS* gene and a poor response to induction therapy at day 7 and prognosis in childhood ALL. The same study indicated an association between higher levels of BCL2 and white blood cell (WBC) count <50,000/mm^3^ at diagnosis and low risk group classification [55]. The differential regulation of pro- and antiapoptotic BCL2 family members appears to be a key event in the execution of dexamethasone-induced apoptosis in ALL cell lines and also indicates a role of these proteins in primary ALL cells [56]. Using primary lymphoblasts from ALL children during systemic glycocorticoid monotherapy and related cell lines, it was shown that a subsequent induction of the proapoptotic BH3 molecules BMF and BIM and also an unexpected significant repression of the proapoptotic BCL2 protein Noxa take place [57]. In addition, a study of the expression of 70 apoptosis genes in relation to lineage, genetic subtype, cellular drug resistance, and outcome in childhood ALL indicated that MCL1 was significantly associated with prednisolone sensitivity, whereas BCL2L13 was correlated with L-asparaginase resistance and with unfavorable clinical outcome [58].

To summarize the most important findings regarding BCL2 gene family in ALL one should note the higher frequency of BCL2 mRNA overexpression and the lower frequency of BAX mRNA overexpression in ALL cases, and the diverse expression of BCL2 in the different types of ALL according to the stage of B-cell maturation. However, different studies have failed to correlate the altered expression of these genes with survival. Findings regarding lineage-dependent BCL2 expression in ALL are controversial. Furthermore, BCL2 levels influence the sensitivity of leukemic cells to therapy and it has been shown that differential regulation of pro- and antiapoptotic BCL2 family members appears to be a key event in the execution of dexamethasone-induced apoptosis in ALL cell lines.

## 4. BCL2 Gene Family and AML

*BCL2* gene family is overexpressed in AML and seems to play an important role not only in disease pathogenesis but also in resistance to chemotherapy. The importance of *BCL2* family members in AML is indicated by the expression of *BCL2, BCL2L12, BCL-XL,* and *BAD* in leukemic CD34+ cells, whereas normal promyelocytes (in non-APL AML cases) (CD34-CD33+) lack *BCL2* and *BCL-XL* expression. A low BCL2/BAX ratio is found in >20% of CD34+ cells, in M0/MI FAB subtypes, and in those patients with poor prognosis karyotype. Leukemic promyelocytes with the phenotype CD34+CD33-CD13—express only BCL-XL protein and not BCL2 [9, 59]. Moreover, the enhanced expression of BCL2 in CD34+ cells offers them a survival advantage and resistance to chemotherapy [59]. Finally, BCL2 expression plays an important role in maintaining a favorable antiapoptotic microenvironment for the survival of AML blasts. In vitro studies show that stable BCL2 protein levels reduce T-cell apoptosis and favour the survival of peripheral blood cells and malignant cells [60]. This microenvironment also prevents T-cell activation and proliferation by inhibition of several molecules like NF-*κ*B, c-MYC, and pRB, that enables malignant cells escape from immune surveillance [61].

BCL2 expression levels have been associated with FAB classification, age, and cytogenetics of AML *in several studies*. BCL2 is not expressed in M2 FAB subtype, in contrast to M4, M5, M6 subtypes. Positive expression of BCL2 is also found in the more immature AML subtypes M0 and M1 [62]. Not only a higher BCL2 expression but also a lower CD95 (or FAS molecule) expression is found in immature FAB M0/M1 AML cells compared to the more mature M2/M5 subtypes. However, no maturation-dependent difference in BAX expression is observed [63]. On the other hand, Kornblau et al. [64] found no association between BCL2 expression and FAB classification, the percentage of blasts or cytogenetic abnormalities.

Cytogenetics is the most important predictive factor in AML and the association of apoptosis and several gene mutations or chromosomal abnormalities is interesting. The prognostically favorable chromosomal translocation t(8;21), which is commonly found in middle-aged adult AML patients, creates the AML1/ETO fusion protein and induces antiapoptotic BCL2 expression in vitro [65], but this is not confirmed in vivo [66]. Additionally, high BCL2 protein levels were detected by Western blotting in 198 patients with AML and were considered to be an adverse prognostic factor for patients with favorable or intermediate prognosis cytogenetics, for example, inversion (16), t(8;21), t(15;17). On the contrary, high BCL2 levels represent paradoxically a favorable prognostic factor for the group of patients with poor risk karyotype (e.g., 11q23, Ph+, deletion 5 and 7, or complex changes) [64]. Several studies indicate that BCL2 is a prognostic factor for AML [67]. Patients with higher BCL2 mRNA levels show lower complete remission (CR) rates and worse outcome. There is no association between remission rate or survival and BCL2 expression in patients >60 years and in patients with AML following myelodysplastic syndromes [67]. As far as other members of the BCL2 family proteins are concerned, high levels of BAD and BAX mRNA are associated with patient failure to enter CR and increased BAD or BAD and BAX expression predicted an adverse outcome regardless of the response to induction chemotherapy. Following induction chemotherapy, the presence of increased levels of BAX and BCL2/BAX ratio are independent predictors of unfavorable outcome [68]. In contrast, Ong et al. found that high BAX expression at diagnosis is correlated with significantly longer disease-free survival, event-free survival, and overall survival [69]. Abnormal expression profile of BCL-X gene is associated with recurrence in AML, but no mutation in BCL-X gene has been detected. There are two products of this gene, BCL-XL and BCL-XS. BCL-XL transcript is found in most patients at diagnosis and during relapse, but BCL-XS transcript is detected in fewer cases. There is an indication that the loss of BCL-XS expression is a prognostic factor in AML, but this requires further investigation [70]. 

Correlation of BCL2 family members with other proteins that appear to influence their levels of expression is also interesting. Protein kinase C (PKC) phosphorylates BCL2 protein and BAX modulates BCL2 dimerization. It was found that, in AML patients, BAX and PKCa levels are heterogeneous, do not correlate with the percentage of blasts in the sample, and their expression is similar among FAB groups with a greater range for M4. Patients with inversion 16 had lower BAX levels. No correlation with prognosis was found. Nevertheless, low BCL2/BAX and PKCaB2/BAX ratios correlate with longer survival. Patients with unfavorable cytogenetics are an exception to this finding and have the worst outcome [71].

A novel receptor tyrosine kinase, named AXL, was found to be expressed in AML specifically of monocytic origin. Thirty-five percent of AML patients express this kinase. CD34+ cells show high expression levels of both BCL2 and AXL, suggesting a possible correlation between the two proteins. No difference in prognosis between patients positive or negative for AXL expression is found, but patients with very high levels of this protein have a dismal outcome [72].

Additionally, BCL2 expression is subjective to cytokines. Blasts produce high interleukin-1 (IL-1) levels, in the absence of exogenous growth factors. IL-1 enhances the autonomous growth of these cells [73]. Activation of IL-1 receptor leads to leukemic cell survival and poor outcome through three signaling pathways. The first is PIK3 pathway and interferes with the BCL2 protein family: it either activates PKC and then BCL2 via phosphorylation, or activates pAkt which subsequently inactivates BAD through phosphorylation [74, 75]. On the other hand, there are some cytokines, such as interferon-*γ* [76], epidermal growth factor (EGF) and granulocyte-macrophage colony-stimulating factor (GM-CSF) that, under certain circumstances, have dual function by inducing not only antiapoptotic but also pro-apoptotic signals. For example, GM-CSF act by phosphorylating STAT-5, upregulating cyclin D and stimulating cell proliferation. It can also upregulate procaspase 3 levels and activate caspase 3, cleave PARP, upregulate Jak-STAT-dependent pro-apoptotic proteins like BAX, BCL2, BCL-XL, and XIAP and therefore induce cell death [77].

**Figure 2:**
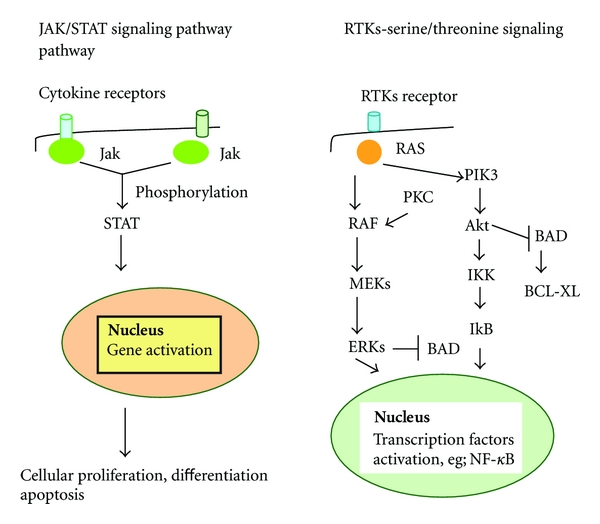
Cellular signaling pathways that control normal hematopoiesis and apoptosis. Molecules participating in these pathways could be used as therapy targets in leukemias. Jak/Stat pathway is very important in leukemias, since disorders of its function lead to malignancies, for example, chromosomal translocation TEL-Jak2. It participates in apoptosis regulation with various ways, for instance some Stats (2 and 3) are the mediators of the antiapoptotic effects of cytokines, like IL-6 and IL-2. RTKs are membrane-bound enzymes that phosphorylate and activate several signaling proteins. Example of this receptor is the FLT3R and mutations of the FLT3 gene have been reported in some cases of AML. Other molecules like ERKs and Akt are involved in apoptosis through suppressing bad mediated apoptosis. Jak: Janus kinase. STAT: signal transducer and activator of transcription. MEKs: MAPK kinases. MAPK: mitogen-activated protein kinase. ERKs: extracellular signal-regulated kinases. RTK: receptor tyrosine kinase. PKC: protein kinase C. FLT3R: FMS-like tyrosine kinase 3 receptor. Akt: protein kinase B (PKB). PIK3: phosphatidylinositol 3-kinase. IkB: inhibitor of nuclear factor kB. NF-*κ*B: nuclear factor kB.

Another growth promoting pathway with prognostic value in AML is MEK/MAPK pathway, which is associated with an apoptosis-resistance phenotype due to its antiapoptotic function. It is found that in primary AML cells, MAPK is constitutively active and promotes leukemic growth and survival. Therefore, patients with low antiapoptotic BAX/BCL2 ratio and constitutively active MAPK pathway have a poor prognosis, because these two factors synergistically act and enhance leukemic cell survival (Figure 2) [78].

Summarizing the most important findings concerning BCL2 family of genes in AML, BCL2 gene family is overexpressed in AML and seems to play an important role not only in disease pathogenesis, but also in resistance to chemotherapy. BCL2 expression levels have been associated in different studies with FAB classification, age, and cytogenetics of AML. Additionally, high BCL2 protein levels were considered to be an adverse prognostic factor for patients with favorable or intermediate prognosis cytogenetics and paradoxically a favorable prognostic factor for the group of patients with poor risk karyotype. As far as other members of the BCL2 family are concerned, high levels of BAD and BAX mRNA are associated with patient failure to enter CR while increased BAD or BAD and BAX expression predicted an adverse outcome regardless of the response to induction chemotherapy. The correlation of BCL2 family members with other proteins that influence their levels of expression, such as (PKC), AXL, cytokines, or MEK/MAPK pathway, is also interesting.

## 5. BCL2 Family of Genes and CLL

B-cell Chronic Lymphocytic Leukemia (CLL) is characterized by the accumulation of malignant clonal CD5+ CD23+ B cells. 

The most common chromosomal abnormalities in CLL are 13 (13q14) and inversion t(11; 14)(q13; q32). Exertions at long arm of chromosome 18 (18q21) (q32) lead to BCL2 oncogene activation, while the inversion t(14; 19) (q32q13.1) activates the BCL-3 oncogene [79].

Malignant CLL B cells overexpress BCL2. Until 2005 no mechanism had been discovered to explain BCL2 deregulation in CLL, with the exception of <5% of cases in which the BCL2 gene is juxtaposed to Ig loci. Interestingly, over the last few years, the importance of microRNAs (miRNAs) came to the frontline. These are short noncoding RNAs of *≈*19–24 nt, that regulate gene expression by imperfect base pairing with complementary sequences located mainly, but not exclusively, in the 3′ UTRs of target mRNAs. miRNAs represent one of the major regulatory gene families in eukaryotic cells by inducing translational repression and transcript degradation. The miR-15a and miR-16-1 are located in a cluster at 13q14.3, a genomic region which is frequently deleted in CLL. Deletions and translocations involving these two miRNAs, as well as their downregulation, were found in 65% of B cells in CLL patients. Cimmino et al. demonstrated in 2005 that miR-15a and miR-16-1 expression is inversely related to BCL2 expression in CLL and that both miRNAs negatively regulate BCL2 at a posttranscriptional level. Therefore, miR-15a and miR-16-1 are natural antisense BCL2 interactors that could be used for therapy of BCL2 overexpressing tumors [80, 81].

Furthermore, various studies have focused on the impact of single nuclueotide polymorphisms (SNPs) of the BCL2 family genes in CLL. The polymorphism 938C > A within an inhibitory region of the BCL2 promoter has been reported to regulate BCL2 protein expression and to be associated with adverse prognostic features in CLL (shorter overall survival, time to first treatment, disease stage at diagnosis and ZAP-70 status) [82]. Nevertheless, more recent studies have not confirmed the association of this SNP with BCL2 protein levels or with any clinical or laboratory parameters [83]. Concerning the other genes of BCL2 family, studies about the prognostic role of the polymorphism G(-248)A in the promoter region of the BAX gene are contradictive [84, 85], whereas an SNP in the MCL-1 promoter region has been shown to characterize CLL patients at high risk of relapse [86].

Finally, the role of epigenetic alterations is under investigation in CLL. In the majority of patients, the promoter region for BCL2 is hypomethylated, which may contribute to increased transcription and BCL2 protein expression in CLL [87].

Despite the findings from in vitro assays, not all clinical studies have identified an association between BCL2 family members expression and patients' outcome in B-CLL. There are several studies showing no correlation between BCL2 protein expression and clinical features like age, sex, Rai stage, platelet count, Hb concentration, and lymph node involvement [88, 89] or with disease prognosis [90–92]. However, Faderl et al. [93] used a large sample of patients (230) and RIA as method for protein detection and found an association between BCL2, cyclin D1, FAS, PCNA, ATM and patients' survival. They also suggested that BCL2 is the most important protein in predicting survival among studied proteins, since marked elevation of BCL2 was linked to worst outcome. Another assay [89] showed elevated expression of BCL2, MCL-1, BAG -1, BAX, BAK, and caspase 3 in contrast to absence of BCL-XL and BAD expression in cells from 58 patients with CLL. This study also indicated that higher levels of MCL-1 were associated with resistance to chemotherapy; higher levels of BAG-1 correlated with marginal failure to achieve complete remission; and high levels of BCL2 expression and a high BCL2/BAX ratio were associated with elevated WBC. Moreover, a study by Faria et al. that evaluated BCL2 protein levels before and after treatment with fludarabine indicated increased MCL1 and BAG-1 expression in fludarabine-resistant cells. Therefore, it could be assumed that BAG-1 expression might identify CLL patients who will need treatment earlier [94].

BCL2 family members have also been linked to resistance to chemotherapy in CLL. More specifically, BFL1 mRNA levels were inversely correlated with the apoptotic response to in vitro fludarabine treatment of B-CLL cells [95, 96]. In addition, an in vitro study of Pepper et al. [84] regarding resistance of CLL cells to chlorambucil treatment linked high levels of BCL2 and low levels of BAX protein expression to chemoresistance. It was observed that cells undergoing apoptosis demonstrated a remarkable elevation of BAX protein. Therefore, BAX was suggested as a critical protein in determining apoptosis in leukemic cells [91, 97]. Beyond BAX protein, MCL-1 and BAG-1 seem to be very important in apoptosis resistance. MCL-1 is associated with dismal prognosis [97], whereas BAG-1 protein is a very important protein involved in apoptosis resistance, since its expression is very high in viable cells after fludarabine incubation [94].

The relationship between BCL2 and other proteins in CLL is also interesting. BCL2 and p53 expression seem to be inversely related. There are indications that P53 protein overexpression downregulates BCL2 expression in a subgroup of B-CLL patients, but this assumption needs further investigation [98]. Another interesting point is that BCL2 is often overexpressed early in the course of the disease, whereas P53 is found in advanced stages [99]. However, P53 mRNA expression is similar between B-CLL cells and normal cells, while P53 overexpression is considered the result of posttranscriptional modification [91]. 

As far as the relationship of BCL2 and cytokines is concerned, interleukin-4 and interferon-*γ* are found to protect CLL cells from apoptosis, since high levels of IL-1 are associated with low cellular expression of BCL2 protein [100]. NF-*κ*B is also found to inhibit apoptosis. NF-*κ*B is a dimeric nuclear transcription factor. CLL cells exhibit high levels of NF-*κ*B compared to normal cells [101], and in vitro death of B-CLL cells is accompanied with the loss of NF-*κ*B and PI3K/AKT activities [102]. Moreover, specific inhibition of Akt induced extensive apoptosis of CLL cells, which was associated with both a rapid loss of MCL1 through proteasomal degradation and increased expression of p53. CLL clones consistently contain activated Akt which plays a pivotal role in maintaining cell survival. Inhibition of the Akt pathway may be of potential value as a novel therapeutic strategy in the disease [103]. These examples show that the interaction of apoptosis proteins with factors like cytokines or transcription factors involved in pathogenesis of leukemias is important for the understanding of the complex biology of these malignancies.

To summarize the most important findings concerning the role of BCL2 family in CLL, we should highlight that malignant CLL B cells overexpress BCL2, possibly through downregulation of miR-15a and miR-16-1. These are located in a genomic region which is frequently deleted in CLL. miR-15a and miR-16-1 expression is inversely related to BCL2 expression in CLL and both miRNAs negatively regulate BCL2 at a posttranscriptional level. The role of epigenetic alterations is also under investigation in CLL. In the majority of patients, the promoter region for BCL2 is hypomethylated, which may contribute to increased transcription and BCL2 protein expression. Important findings of other studies include a link of marked elevation of BCL2 to worst outcome, association of higher levels of MCL-1 with resistance to chemotherapy and association of higher levels of BAG-1 with failure to achieve complete remission. Moreover, BCL2 family members have been linked to resistance to chemotherapy of CLL patients (Table 1).

## 6. BCL2 Gene Family and CML

BCR/ABL affects a number of molecular pathways, including apoptosis. Dysregulation of the expression of BCL2 protein seems to play a role in disease progression as shown in mouse models. It is overexpressed in CML cells and acts synergistically with BCR/ABL in inducing blast crisis. BCL2 is more important than c-MYC or RAS oncoproteins in the transformation of chronic to blastic phase [104]. In vivo studies showed that BCL2 expression is restricted to the lymphocyte and blast subpopulation cells at the chronic phase, whereas it is higher in the accelerated and the blastic phase. However, other in vivo studies show that c-MYC is more important than BCL2 protein in disease progression since (a) it is expressed in more immature cells, (b) its unregulated expression can inhibit myeloid differentiation, and (c) its levels are increased in the peripheral blood blast cell subpopulation in the accelerated and blastic phase [105, 106]. These studies also suggest that the expression of apoptosis oncoproteins such as BCL2, BAX, FAS and caspase-3 is not associated with the three different phases of CML, since no phase-related predominance of their levels was found.

BCR/ABL activates several signaling pathways, such as PIK3, STAT, Ras, and NF-*κβ* which influence the expression of members of the BCL2 protein family. There are several examples of this: AKT is a serine/threonine kinase which regulates survival signals in response to several internal and external signals. It becomes activated through the PIK-3 signaling pathway and its role focuses on inhibiting cell death by two ways: the first is the inactivation of pro-apoptotic proteins like BAX or caspase-9, and the second is the inactivation of NF-*κβ* and eventually BCL-XL. Another example is STAT proteins, which are cytoplasmic proteins activated by phosphorylation after recruitment to an activated receptor complex. When active STAT proteins translocate to the nucleus, they bind to specific DNA response elements and induce the expression of STAT-regulated genes. The latter play an important role in haemopoiesis and in heamopoietic cell function, such as in Th1 or Th2 response in lymphocyte function. STAT 1, 3, and 5 control cell cycle and apoptosis and STAT 5 is found constitutively active in CML patients [107]. The fusion BCR/ABL tyrosine kinase activates the PIK-3/AKT pathway and the result is either phosphorylation of BAD protein at its serine residues, or enhanced expression of BCL-XL protein. Overexpression of BCL-XL can be accomplished through the activation of the STAT-5 protein. BAD and BCL-XL are considered to be the most important regulators of apoptosis in CML [108, 109]. 

It has also been shown that high levels of BCR/ABL expression are responsible for the prevention of the early translocation of the pro-apoptotic proteins BAD and BAX from the cytosol to the mitochondrion following a cytotoxic signal, explaining the resistance of cells that express high BCR/ABL levels to cytotoxic drugs [110]. 

Another member of the BCL2 superfamily, the BH3-only pro-apoptotic protein BIM is considered to be an important target in CML cells, as its downregulation is associated to the survival of leukemic cells. It is shown in mouse models that BIM is an essential cytokine-dependent regulator of normal haemopoiesis [111]. BCR/ABL tyrosine kinase reverses the induction of BIM mRNA caused by cytokine deprivation in hematopoietic progenitor cells, and it also down-regulates BIM expression in human CML cell lines. Therefore BIM is considered to be an important downstream target in CML cells that express BCR/ABL. The most likely pathway involved in BIM mRNA down-regulation by BCR/ABL is considered to be PIK-3 pathway [112].

Antiapoptotic protein MCL-1 is reported to be another interesting target in CML, since BCR/ABL expressing cells show higher expression of MCL-1 mRNA and MCL-1 protein, and the use of BCR/ABL inhibitor imatinib results in the decrease of MCL-1 expression in CML cell lines [113].

In summary, in CML BCR/ABL is the key regulating mechanism in disease pathogenesis and affects many signaling pathways including apoptosis. Nevertheless, BCL2 family proteins do not seem to play a determining role in CML.

## 7. BCL2 Family Proteins and Leukemia Treatment

Since BCL2 family proteins are pivotal regulators of apoptotic cell death and given their deregulation in acute and chronic leukemias, the concept of manipulating their function towards enhancing their antitumor effects seems a reasonable strategy in the design of antileukemic therapeutic agents.

Impaired apoptosis reated to overexpression of BCL2 protein, which is observed in approximately 76% of patients with CLL, is implicated in the resistance to chemotherapy. BCL2 antisense oligonucleotides like oblimersen are used in order to downregulate BCL2 oncoprotein in several hematopoietic malignancies, including CLL, multiple myeloma and non-Hodgkin lymphoma [114–116]. In order to achieve better clinical results, these agents are used in combination with traditional drugs like fludarabine or cyclophosphamide and monoclonal antibodies like rituximab in several clinical trials. While the combination of chemotherapeutic agents (fludarabine, cyclophosphamide and rituximab) has good results in patients with CLL [117], the addition of BCL2 antisense oligonucleotides appears promising [118]. 

Oblimersen has also been administered during induction and consolidation treatment in untreated elderly AML patients. After 72-hour oblimersen infusion, Bcl-2/ABL mRNA copies were decreased compared with baseline in patients that achieved CR, whereas it was increased in nonresponders. Changes in Bcl-2 protein showed a similar trend. The degree of Bcl-2 downregulation may correlate with the response to therapy [119]. Another study demonstrated that oblimersen can also be administered safely with FLAG chemotherapy, downregulating its target, Bcl-2, in previously untreated high-risk AML patients (i.e., age at least 60 years) [120, 121]. Furthermore, expression levels of BCL2 and BCL2L12 were found to be significantly altered during apoptosis induced by widely used chemotherapeutic drugs in human leukemia cells, supporting their usefulness as biomarkers to predict response to therapy [9, 14, 122–128]. 

Studies investigating BCL2 family inhibitors, such as ABT-737 and ABT-263, in ALL are really interesting. ABT-737 is a pan-BCL2 inhibitor that has a wide range of single agent activity against ALL cell lines. Furthermore, ABT-737 has been shown to enhance the activity of vincristine, L-Asparaginase and dexamethasone against ALL cells. On the other hand, ABT-263 is a potent, orally bioavailable BAD-like BH3 mimetic that induces complete tumor regressions in xenograft models of ALL. A relationship between MCL1 expression and resistance to ABT-737 has been reported, which is abolished with the use of a synthetic cytotoxic retinoid, N-(4-hydroxyphenyl) retinamide, that phosphorylates and inhibits MCL1 [129–132].

Interestingly, CLL cells were previously reported to be highly sensitive to BCL2 inhibition and treatment with ABT-737. Moreover, recent data support that, although structurally similar and exhibiting similar binding affinities to antiapoptotic BCL2 family proteins, ABT-263 is less potent than ABT-737 in inducing apoptosis in CLL cells. Furthermore, binding of ABT-263 to albumin seems to markedly increase the concentration of drug required to induce apoptosis and clear CLL cells from the blood in vivo [133–138].

The use of BCL2 inhibitors in the treatment of leukemias is promising, either alone or in combination with classical treatments. Given the fact that therapeutic interventions in leukemias are often not adequate nor successful, new therapeutic plans are more than welcome.

## 8. Conclusions

BCL2 protein family plays an important role in regulating the cellular program of apoptosis. Normal cellular homeostasis appears to be dependent on the balance between pro- and antiapoptotic members of BCL2 family. BCL2 is overexpressed in almost all types and subtypes of leukemia, indicating the importance of this molecule in disease pathogenesis and evolution. BCL2 is the most well studied member of the family, but evidence shows that other BCL2 related family proteins like BAX and MCL-1 are important as well. More specifically, MCL-1 is related to almost all leukemias that show resistance to chemotherapy and bad prognosis. Expression levels of *BCL2* and *BCL2L12* were altered during apoptosis induced by widely used chemotherapeutic drugs in human leukemia cells. These molecules may not be used as disease markers in most cases, but their importance lies in (a) explaining drug chemoresistance and (b) in the effort to design new agents with a greater specificity. Further research should focus on the role of BCL2 family members in leukemogenesis. It is clear that clinical studies are only beginning to assess the expression of BCL2 family members. Further research is more than valuable in the understanding of the importance of this gene family in leukemias.

## Figures and Tables

**Table 1:** This table summarizes the general findings regarding BCL2 family members in each type of leukemia.

Type of Leukemia	BCL2 family members involved in disease	Correlation with overall survival and outcome
ALL	High levels: BCL2, BAX, MCL-1	No correlation
		↑MCL-1 → resistance to chemotherapy

AML	High levels: BCL2, BCL-XL, BAD, BCL2/BAX ratio especially in M4,M5,M6 subtypes and in CD34+ blasts	↑BCL2 and FAS → no correlation
		↑BAD and BAX, ↑BCL2/BAX ratio → worse outcome

CLL	High levels: BCL2, BCL-W, BAD, BAK, BAX, BCL2/BAX ratio	Conflicting results
	No participation of BIK and BCL-XL	↑MCL-1, ↑BAX, ↑BAG-1 ↑BCL2 → resistance to chemotherapy

CML	High levels: MCL-1, BCL2 Low levels of BIM	BCL2: key protein in disease progression

**Table 2:** This table summarizes the properties of the most well-studied members of BCL2 family.

	Action	Mechanism of action	Subcellular localization
BCL2	Antiapoptotic	Inhibits apoptosis by preservation of mitochondrial membrane integrity	(i) Outer mitochondrial membrane
			(ii) Nuclear envelope
			(iii) Membrane of the endoplastic reticulum (ER)

BCL-XL	Antiapoptotic	Inhibits cytochrome c release through the mitochondrial pore that inhibits activation of the cytoplasmic caspase cascade by cytochrome c	Transmembrane molecule in the mitochondria

BCL-W	Antiapoptotic	Reduced cell apoptosis under cytotoxic conditions	Exclusively on the mitochondrion

MCL-1	Antiapoptotic	Short half-life, interaction with BAK1, Noxa, BCL2L11, Bcl-2-associated death promoter, PCNA	Mitochondria, nucleus

BAX	Proapoptotic	Release of apoptogenic factors like cytochrome c, activation of caspase cascade	Cytosol

BAK	Proapoptotic	Undergoes conformational changes to form larger aggregates during apoptosis	Integral mitochondrial membrane protein

BID	Proapoptotic	Direct activator of Bax	Cytosol and membrane

BIM	Proapoptotic	Free Bim binds to Bcl-2 or Bcl-XL and inactivates their antiapoptotic functions	Free BIM in mitochondria

BAD	Proapoptotic	Dephosphorylated BAD forms a heterodimer with Bcl-2 and Bcl-xL, inactivating them and thus allowing Bax/Bak-triggered apoptosis	Free BAD in mitochondria
